# 2-(Hy­droxy­meth­yl)pyridin-3-ol

**DOI:** 10.1107/S1600536812002759

**Published:** 2012-02-17

**Authors:** Xue-Jun He, Jiang-Kai Qiu, Yu-Chi Jia, De-Cai Wang, Ping-Kai Ou-Yang

**Affiliations:** a State Key Laboratory of Materials-Oriented Chemical Engineering, School of Pharmaceutical Sciences, Nanjing University of Technology, Nanjing 210009, People’s Republic of China

## Abstract

In the crystal structure of the title compound, C_6_H_7_NO_2_, the mol­ecules are are linked by inter­molecular O—H⋯N and O—H⋯O hydrogen bonds; π–π stacking is observed between parallel pyridine rings of adjacent mol­ecules [centroid-to-centroid distance = 3.7649 (12) Å].

## Related literature

For the synthesis of the title compound, see: Dabak (2002 ▶).
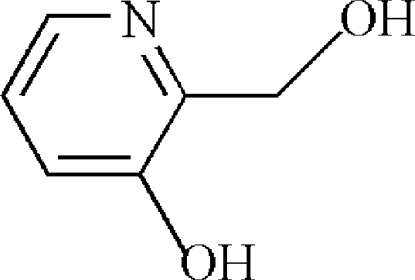


## Experimental

### 

#### Crystal data

C_6_H_7_NO_2_
*M* *_r_* = 125.13
Monoclinic, 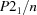

*a* = 7.0430 (14) Å
*b* = 7.1280 (14) Å
*c* = 12.264 (3) Å
β = 100.30 (3)°
*V* = 605.8 (2) Å^3^
*Z* = 4
Mo *K*α radiation
μ = 0.10 mm^−1^
*T* = 293 K
0.30 × 0.20 × 0.10 mm

#### Data collection

Enraf–Nonius CAD-4 diffractometer
Absorption correction: ψ scan (*XCAD4*; Harms & Wocadlo, 1995 ▶) *T*_min_ = 0.969, *T*_max_ = 0.990
2263 measured reflections
1089 independent reflections
932 reflections with *I* > 2σ(*I*)
*R*_int_ = 0.028
3 standard reflections every 200 reflections intensity decay: 1%

#### Refinement

*R*[*F* ^2^ > 2σ(*F* ^2^)] = 0.036
*wR*(*F* ^2^) = 0.136
*S* = 0.99
1089 reflections
85 parameters
H-atom parameters constrained
Δρ_max_ = 0.18 e Å^−3^
Δρ_min_ = −0.15 e Å^−3^

### 

Data collection: *CAD-4 EXPRESS* (Enraf–Nonius, 1994 ▶); cell refinement: *CAD-4 EXPRESS*; data reduction: *XCAD4* (Harms & Wocadlo, 1995 ▶); program(s) used to solve structure: *SHELXTL* (Sheldrick, 2008 ▶); program(s) used to refine structure: *SHELXTL*; molecular graphics: *SHELXTL*; software used to prepare material for publication: *SHELXTL*.

## Supplementary Material

Crystal structure: contains datablock(s) I, global. DOI: 10.1107/S1600536812002759/xu5437sup1.cif

Structure factors: contains datablock(s) I. DOI: 10.1107/S1600536812002759/xu5437Isup2.hkl

Supplementary material file. DOI: 10.1107/S1600536812002759/xu5437Isup3.cml

Additional supplementary materials:  crystallographic information; 3D view; checkCIF report

## Figures and Tables

**Table 1:** Hydrogen-bond geometry (Å, °)

*D*—H⋯*A*	*D*—H	H⋯*A*	*D*⋯*A*	*D*—H⋯*A*
O1—H1*A*⋯O2^i^	0.82	1.85	2.6502 (17)	166
O2—H2*A*⋯N^ii^	0.82	1.92	2.7216 (17)	167

Symmetry codes: (i) 
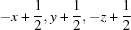
; (ii) 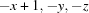.

**Fig. 1.**
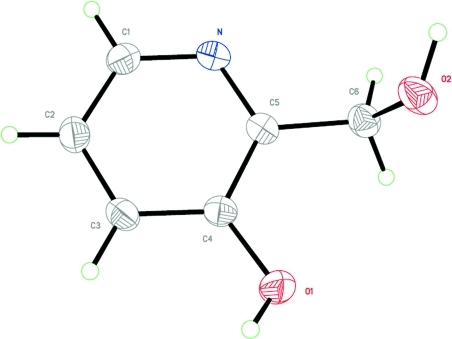
The structure of the title compound, showing the atomic numbering scheme. Non-H atoms are shown with 30% probability displacement ellipsoids.

## supplementary crystallographic information

### Comment

The title compound is an important organic intermediate for the synthesis of 2-pyrimidine-oxy-*N*-aryl benzyl amine derivatives, an important compound for new pesticides. In the process of synthesis, we obtained the crystal of the intermediate and we report its crystal structure.

As illustrated in Fig. 1, the hydroxyl oxygen O1 and the hydroxymethyl carbon C6 are approximately coplanar with the pyridine ring (C1—C5/N) with the maximum deviation of -0.0227 Å. The crystal structure is stabilized by intermolecular N—H···O and O—H···O hydrogen bonds (Table 1), and is further stabilized by π–π stacking between pyridine rings [centroid–centroid distance = 3.7649 (12) Å]

### Experimental

The synthesis is according to the literature (Dabak, 2002). The formaldehyde solution (12.6 ml, 0.156 mol) and sodium hydroxide (6.3 g, 0.158 mol) was added to a solution of 3-hydroxypyridine (15.0 g, 0.156 mol) in water (63 ml). The reaction mixture was heated at 373 K for 12 h and then allowed to cool to ambient temperature. Acetic acid (9.47 ml, 0.156 mol) was added and water was removed *in vacuo* and the solid obtained was stirred with acetone (200 ml). The extract was purified by silica gel column chromatography and the colourless crystals were obtained in a yield of 20.3%.

### Refinement

H atoms were placed at calculated positions and were treated in riding mode with C—H = 0.93 (aromatic), 0.97 Å (methylene) and O—H = 0.82 Å. *U*_iso_(H) = 1.2*U*_eq_(C) and 1.5*U*_eq_(O).

### Crystal data

**Table:**

C_6_H_7_NO_2_	*F*(000) = 264
*M**_r_* = 125.13	*D*_x_ = 1.372 Mg m^−^^3^
Monoclinic, *P*2_1_/*n*	Mo *K*α radiation, λ = 0.71073 Å
Hall symbol: -P 2yn	Cell parameters from 25 reflections
*a* = 7.0430 (14) Å	θ = 9–13°
*b* = 7.1280 (14) Å	µ = 0.10 mm^−^^1^
*c* = 12.264 (3) Å	*T* = 293 K
β = 100.30 (3)°	Block, colourless
*V* = 605.8 (2) Å^3^	0.30 × 0.20 × 0.10 mm
*Z* = 4	

### Data collection

**Table:**

Enraf–Nonius CAD-4 diffractometer	932 reflections with *I* > 2σ(*I*)
Radiation source: fine-focus sealed tube	*R*_int_ = 0.028
Graphite monochromator	θ_max_ = 25.2°, θ_min_ = 3.1°
ω/2θ scans	*h* = 0→8
Absorption correction: ψ scan (*XCAD4*; Harms & Wocadlo, 1995)	*k* = −8→8
*T*_min_ = 0.969, *T*_max_ = 0.990	*l* = −14→14
2263 measured reflections	3 standard reflections every 200 reflections
1089 independent reflections	intensity decay: 1%

### Refinement

**Table:**

Refinement on *F*^2^	Secondary atom site location: difference Fourier map
Least-squares matrix: full	Hydrogen site location: inferred from neighbouring sites
*R*[*F*^2^ > 2σ(*F*^2^)] = 0.036	H-atom parameters constrained
*wR*(*F*^2^) = 0.136	*w* = 1/[σ^2^(*F*_o_^2^) + (0.1*P*)^2^ + 0.069*P*] where *P* = (*F*_o_^2^ + 2*F*_c_^2^)/3
*S* = 0.99	(Δ/σ)_max_ < 0.001
1089 reflections	Δρ_max_ = 0.18 e Å^−^^3^
85 parameters	Δρ_min_ = −0.15 e Å^−^^3^
0 restraints	Extinction correction: *SHELXTL* (Sheldrick, 2008), Fc^*^=kFc[1+0.001xFc^2^λ^3^/sin(2θ)]^-1/4^
Primary atom site location: structure-invariant direct methods	Extinction coefficient: 0.67 (5)

### Special details

**Table:**

Geometry. All e.s.d.'s (except the e.s.d. in the dihedral angle between two l.s. planes) are estimated using the full covariance matrix. The cell e.s.d.'s are taken into account individually in the estimation of e.s.d.'s in distances, angles and torsion angles; correlations between e.s.d.'s in cell parameters are only used when they are defined by crystal symmetry. An approximate (isotropic) treatment of cell e.s.d.'s is used for estimating e.s.d.'s involving l.s. planes.
Refinement. Refinement of *F*^2^ against ALL reflections. The weighted *R*-factor *wR* and goodness of fit *S* are based on *F*^2^, conventional *R*-factors *R* are based on *F*, with *F* set to zero for negative *F*^2^. The threshold expression of *F*^2^ > σ(*F*^2^) is used only for calculating *R*-factors(gt) *etc*. and is not relevant to the choice of reflections for refinement. *R*-factors based on *F*^2^ are statistically about twice as large as those based on *F*, and *R*-factors based on ALL data will be even larger.

### Fractional atomic coordinates and isotropic or equivalent isotropic displacement parameters (Å^2^)

**Table:**

	*x*	*y*	*z*	*U*_iso_*/*U*_eq_	
N	0.59730 (19)	0.28061 (17)	0.05259 (10)	0.0374 (4)	
O1	0.21831 (16)	0.3738 (2)	0.20521 (9)	0.0531 (5)	
H1A	0.2217	0.4225	0.2661	0.080*	
C1	0.7372 (2)	0.3911 (2)	0.10511 (13)	0.0418 (5)	
H1B	0.8548	0.3932	0.0807	0.050*	
O2	0.27768 (19)	−0.02901 (16)	0.08581 (9)	0.0504 (5)	
H2A	0.3277	−0.1099	0.0527	0.076*	
C2	0.7152 (2)	0.5019 (2)	0.19373 (13)	0.0436 (5)	
H2B	0.8160	0.5772	0.2284	0.052*	
C3	0.5409 (2)	0.4995 (2)	0.23031 (13)	0.0410 (5)	
H3A	0.5223	0.5731	0.2901	0.049*	
C4	0.3939 (2)	0.3855 (2)	0.17644 (12)	0.0358 (5)	
C5	0.4270 (2)	0.2764 (2)	0.08702 (11)	0.0340 (5)	
C6	0.2761 (2)	0.1456 (2)	0.02825 (13)	0.0421 (5)	
H6A	0.1500	0.2034	0.0225	0.050*	
H6B	0.2994	0.1228	−0.0462	0.050*	

### Atomic displacement parameters (Å^2^)

**Table:**

	*U*^11^	*U*^22^	*U*^33^	*U*^12^	*U*^13^	*U*^23^
N	0.0432 (8)	0.0343 (7)	0.0360 (7)	0.0046 (5)	0.0108 (5)	0.0008 (5)
O1	0.0435 (8)	0.0704 (9)	0.0487 (8)	−0.0087 (6)	0.0176 (5)	−0.0177 (6)
C1	0.0383 (9)	0.0407 (9)	0.0478 (10)	0.0024 (7)	0.0115 (7)	0.0030 (7)
O2	0.0729 (9)	0.0392 (7)	0.0462 (7)	−0.0084 (6)	0.0299 (6)	−0.0068 (5)
C2	0.0415 (9)	0.0399 (9)	0.0471 (10)	−0.0041 (7)	0.0020 (7)	−0.0038 (6)
C3	0.0462 (9)	0.0392 (8)	0.0377 (9)	0.0008 (7)	0.0076 (7)	−0.0074 (6)
C4	0.0381 (9)	0.0362 (8)	0.0336 (8)	0.0030 (6)	0.0078 (6)	0.0003 (6)
C5	0.0416 (9)	0.0318 (8)	0.0284 (8)	0.0033 (6)	0.0053 (6)	0.0034 (5)
C6	0.0472 (10)	0.0421 (9)	0.0371 (8)	−0.0027 (7)	0.0078 (7)	−0.0049 (6)

### Geometric parameters (Å, º)

**Table:**

N—C1	1.334 (2)	C2—C3	1.381 (2)
N—C5	1.3412 (19)	C2—H2B	0.9300
O1—C4	1.3478 (19)	C3—C4	1.387 (2)
O1—H1A	0.8200	C3—H3A	0.9300
C1—C2	1.375 (2)	C4—C5	1.397 (2)
C1—H1B	0.9300	C5—C6	1.499 (2)
O2—C6	1.430 (2)	C6—H6A	0.9700
O2—H2A	0.8200	C6—H6B	0.9700
			
C1—N—C5	119.08 (12)	O1—C4—C3	123.57 (14)
C4—O1—H1A	109.5	O1—C4—C5	117.43 (13)
N—C1—C2	122.91 (15)	C3—C4—C5	118.99 (15)
N—C1—H1B	118.5	N—C5—C4	121.25 (13)
C2—C1—H1B	118.5	N—C5—C6	117.37 (12)
C6—O2—H2A	109.5	C4—C5—C6	121.35 (14)
C1—C2—C3	118.82 (15)	O2—C6—C5	111.22 (13)
C1—C2—H2B	120.6	O2—C6—H6A	109.4
C3—C2—H2B	120.6	C5—C6—H6A	109.4
C2—C3—C4	118.94 (14)	O2—C6—H6B	109.4
C2—C3—H3A	120.5	C5—C6—H6B	109.4
C4—C3—H3A	120.5	H6A—C6—H6B	108.0
			
C5—N—C1—C2	0.0 (2)	O1—C4—C5—N	179.61 (13)
N—C1—C2—C3	−0.1 (2)	C3—C4—C5—N	−0.1 (2)
C1—C2—C3—C4	0.0 (2)	O1—C4—C5—C6	−2.4 (2)
C2—C3—C4—O1	−179.62 (15)	C3—C4—C5—C6	177.80 (13)
C2—C3—C4—C5	0.1 (2)	N—C5—C6—O2	95.53 (15)
C1—N—C5—C4	0.1 (2)	C4—C5—C6—O2	−82.49 (17)
C1—N—C5—C6	−177.96 (13)		

### Hydrogen-bond geometry (Å, º)

**Table:**

*D*—H···*A*	*D*—H	H···*A*	*D*···*A*	*D*—H···*A*
O1—H1*A*···O2^i^	0.82	1.85	2.6502 (17)	166
O2—H2*A*···N^ii^	0.82	1.92	2.7216 (17)	167

Symmetry codes: (i) −*x*+1/2, *y*+1/2, −*z*+1/2; (ii) −*x*+1, −*y*, −*z*.
